# Phase II study of intravenous etoposide in patients with relapsed ependymoma (CNS 2001 04)

**DOI:** 10.1093/noajnl/vdac053

**Published:** 2022-04-13

**Authors:** John R Apps, Shanna Maycock, David W Ellison, Timothy Jaspan, Timothy A Ritzmann, Donald Macarthur, Conor Mallucci, Keith Wheatley, Gareth J Veal, Richard G Grundy, Susan Picton

**Affiliations:** 1 Cancer Research UK Clinical Trials Unit, University of Birmingham, Birmingham, UK; 2 Department of Pathology, St. Jude Children’s Research Hospital, Memphis, Tennessee, USA; 3 Radiology Department, Nottingham University Hospitals NHS Trust, Nottingham, UK; 4 Children’s Brain Tumour Research Centre, Bio-Discovery Institute and Queen’s Medical Centre, University of Nottingham, Nottingham, UK; 5 Department of Neurosurgery, Alder Hey Children’s NHS Foundation Trust, Liverpool, UK; 6 Newcastle University Centre for Cancer, Newcastle University, Newcastle upon Tyne, UK; 7 Department of Paediatric Oncology, Leeds Children’s Hospital, The Leeds Teaching Hospitals NHS Trust, Leeds, UK

**Keywords:** ependymoma, etoposide, relapse

## Abstract

**Background:**

Relapsed ependymoma has a dismal prognosis, and the role of chemotherapy at relapse remains unclear. This study prospectively evaluated the efficacy of intensive intravenous (IV) etoposide in patients less than 21 years of age with relapsed intracranial ependymoma (NCT00278252).

**Methods:**

This was a single-arm, open-label, phase II trial using Gehan’s two-stage design. Patients received IV etoposide 100 mg/m^2^ on days 1-3, 8-10, and 15-17 of each 28-day cycle, up to maximum of 6 cycles. Primary outcome was radiological response after 3 cycles. Pharmacokinetic analysis was performed in 10 patients.

**Results:**

Twenty-five patients were enrolled and included in the intention-to-treat (ITT) analysis. Three patients were excluded in per-protocol (PP) analysis. After 3 cycles of etoposide, 5 patients (ITT 20%/PP 23%) had a complete response (CR), partial response (PR), or objective response (OR). Nine patients (ITT 36%/PP 41%,) had a best overall response of CR, PR, or OR. 1-year PFS was 24% in ITT and 23% in PP populations. 1-year OS was 56% and 59%, 5-year OS was 20% and 18%, respectively, in ITT and PP populations. Toxicity was predominantly hematological, with 20/25 patients experiencing a grade 3 or higher hematological adverse event.

**Conclusions:**

This study confirms the activity of IV etoposide against relapsed ependymoma, however, this is modest, not sustained, and similar to that with oral etoposide, albeit with increased toxicity. These results confirm the dismal prognosis of this disease, provide a rationale to include etoposide within drug combinations, and highlight the need to develop novel treatments for recurrent ependymoma.

**Key Points**

- Intravenous etoposide has activity in relapsed ependymoma.
- Toxicity is greater than oral etoposide.
- Relapsed ependymoma has poor outcomes.

**Importance of the Study**

Relapsed ependymoma is challenging to manage, has a poor prognosis, and the role of chemotherapy is unclear. In this study, we evaluate intravenous (IV) etoposide and show activity, as evidenced by disease response in up to 41% of patients. However, the response was not sustained and the majority of patients succumbed to their tumor. In no patients did it facilitate definitive surgery and given higher toxicity than oral etoposide it is not recommended as a stand-alone therapy. The results, however, justify the use of IV etoposide within multidrug combinations and highlight the need to develop novel treatment strategies for this disease. Pharmacokinetic data presented are of relevance to other pediatric patients treated with IV etoposide and the molecular profiling further supports the adverse prognosis of those with posterior fossa subtype A tumors with amplification of the chromosome 1q.

Ependymoma is a challenging brain tumor that predominantly affects children and accounts for approximately 7% of all childhood brain tumors.^1,2^ Presently, nearly half of all patients relapse after primary treatment for ependymoma. This usually includes surgery, targeting complete resection, and conformal radiotherapy. At relapse, ependymoma is associated with a poor prognosis with a 5-year survival of around 25%.^3–5^ The majority of patients subsequently suffer multiple relapses with decreasing time intervals between each relapse before finally succumbing to the disease.^5^

Surgery remains the mainstay of treatment, both at initial presentation and relapse. Re-irradiation has been increasingly used, in the absence of a clinical trial, and provides patient benefit with improved duration of remission, but is rarely curative.^5,6^ A trial of focal vs craniospinal re-irradiation has not yet been undertaken. The late effects of significant doses of radiotherapy >100 Gy remain unknown.

A variety of chemotherapy regimens have been used in patients with ependymoma, with a range of response rates.^7–16^ Etoposide is a semi-synthetic glucoside derivative of podophyllotoxin which inhibits topoisomerase II. Widely used in Oncology, it has been included in multidrug combinations used to treat ependymoma, including within the recent Children’s Oncology Group ACNS 0831 (NCT01096368) and International Society of Paediatric Oncology (SIOP) Ependymoma II trials (NCT02265770). At relapse, the role of chemotherapy remains unclear.^2,17^ Single-agent oral etoposide has been used for relapsed ependymoma, with response rates of 40%-50%.^18–21^ There is also current interest in the use of intraventricular etoposide, although this modality is only used to treat leptomeningeal or M1-positive disease, due to limited penetration into tumor or brain parenchyma.^22,23^

To date, there has been no formal evaluation of response to single-agent intravenous (IV) etoposide in ependymoma. Etoposide exhibits a schedule dependency and variable bioavailability that has been demonstrated in many preclinical and clinical studies in other cancer types.^24^ Etoposide is extruded from cells by the multidrug resistance efflux pump (MDR1/PGP), which has been found to be highly expressed within ependymomas, suggesting that combination with MDR1 reversal agents could be beneficial in the future.^25,26^ To advance this concept requires a good understanding of the pharmacokinetics (PK) of IV etoposide administration in this population. CNS 2001 04 was a phase II trial with a primary objective to document the response rate of rapidly scheduled etoposide. The secondary objective was to assess the possibility of second surgery or additional radiotherapy in responding patients.

## Materials and Methods

CNS 2001 04 was a UK Children’s Cancer Study Group (UKCCSG) (now Children’s Cancer and Leukaemia Group [CCLG]) multicenter prospective phase II clinical trial that enrolled patients with histologically proven intracranial ependymoma at first, second, or third relapse after failing conventional treatment. PK sampling was optional and performed in a subset of patients.

### Patient Population

Patients were registered within 2 weeks of imaging confirming relapse, with unequivocal evidence of tumor recurrence or progression by magnetic resonance imaging (MRI) and with measurable enhancing or non-enhancing disease. They were required to have either unresectable disease or to have residual, measurable disease following surgery. Eligibility criteria included: age less than 21 years, an expected survival of at least 8 weeks, absolute neutrophil count (ANC) >1.0 × 10^9^/L, platelet count >100 × 10^9^/L, serum total bilirubin lower than the upper limit of laboratory normal (ULN) and aspartate transaminase (AST) < 2 × ULN.

Patients were excluded if surgery alone was likely to achieve a complete resection, they had an unrelated medical condition which would render them unable to receive chemotherapy, had active infection, or were known to be human immunodeficiency virus (HIV)-positive. Patients were not excluded if they had received prior chemotherapy treatment for their tumor, including IV etoposide, though a minimum of 4 weeks must have elapsed since prior chemotherapy and 6 weeks from radiotherapy before initiation of trial treatment.

Written informed consent was obtained from all patients, parents, or legal guardians as appropriate. National and local regulatory and ethical approvals were obtained according to national regulations. The trial was registered on ClinicalTrials.gov (NCT00278252).

### Study Treatment

Patients received up to 6 courses of IV etoposide. Each course lasted 28 days and involved administration of IV etoposide 100 mg/m^2^ over 1 hour at a concentration of 0.24-0.40 mg/mL for 3 consecutive days on 1-3, 8-10, and 15-17. The course was then repeated on day 28 or at count recovery. If neutrophils were <1 × 10^9^/L or platelets <100 × 10^9^/L at the start of the next course, this was postponed until neutrophils were >1 × 10^9^/L and platelets >100 × 10^9^/L. If the criteria for blood count recovery was not been met by day 35, the dose of the subsequent courses was reduced by 25% on each day of etoposide. Further dose reductions were similarly carried out if blood count recovery was once again delayed beyond day 35. MRI was performed after 2 courses. If tumor progression had not occurred, 1 further course was given and imaging was repeated. If the neurosurgical opinion was that resection was possible, chemotherapy was discontinued, and the patient proceeded to surgery. If there was evidence of progressive disease, no further courses were administered. In the absence of tumor progression after 3 courses of etoposide, treatment could be continued to a maximum of 6 courses. If the tumor was deemed resectable at this time, the surgery took place. If radiotherapy was felt appropriate by the treating clinicians, this was performed.

### Disease Evaluation

Imaging was performed according to UKCCSG/French Society of Paediatric Oncology (SFOP) radiological guidelines at the time of the study.^27^ Baseline imaging with three-dimensional tumor assessment by MRI was performed within 2 weeks prior to initiation of etoposide. SIOP definitions of response were used: Complete response (CR)—no radiological evidence of tumor on contrast-enhanced CT or MRI scan. Partial response (PR)—greater than 50% reduction of the product of 2 maximum perpendicular diameters of the tumor relative to the baseline evaluation. Objective response (OR)—reduction in the size of all unequivocal residual tumor manifestations by between 25% and 50% radiographically. There should be no tumor progression and no evidence of new tumor lesions. Stable disease (SD)—less than 25% reduction of the product of 2 maximum perpendicular diameters of the tumor relative to the baseline evaluation without radiological evidence of tumor progression or dissemination. Progressive disease (PD)—any radiological or clinical evidence of tumor progression.^28^ Progressive neurological deterioration even in the face of an unchanged tumor volume, as determined by contrast-enhanced brain studies (CT or MRI), was considered as PD.

Pathological slides and radiology images were also centrally reviewed by the study pathologist or radiologist, where available this was used in preference to local report.^29^ Toxicity was assessed according to the common toxicity criteria (CTC) following each course of chemotherapy.

### Molecular Analysis

Ethical approval was obtained for retrospective analyses of ependymoma specimens (05/MRE04/70, protocol number: 2000 BS 06(b)). All tissues were stored and analyzed according to the UK Human Tissue Act. DNA was extracted from paraffin-embedded samples using the AllPrep FFPE DNA/RNA extraction kit (Qiagen). DNA methylation profiles were generated and assigned as previously described.^5^ Classifier scores ranged from 0.98 to 1, indicating high correlations with the reference dataset. 1q status was evaluated by fluorescent in-situ hybridization (FISH) and/or multiplex ligation-dependent probe amplification (MLPA) as previously described.^5^

### Study Design and Statistical analysis

The trial was a single-arm, open-label, phase II trial using Gehan’s two-stage design with a target response rate of 20% and 5% alpha. The sample size for stage 1 was 14, while stage 2 sample size was dependent on stage 1 observed responses, with a maximum of 11 additional patients if there were 4 or more responses in stage 1. The primary outcome was response after cycle 3 of treatment. Secondary outcome measures included: best overall response during treatment, overall survival (OS) defined as time from trial entry to death from any cause; progression-free survival (PFS) defined as time from trial entry to progression or death from any cause, incidence of radiotherapy and incidence of surgery.

Outcomes have been analyzed descriptively. Survival outcomes (OS and PFS) have been calculated using the Kaplan-Meier method. Confidence intervals for binary outcomes have been calculated using Wilson’s method. All analysis was performed using R 4.1.0.^30^ The final data snapshot was taken on June 10, 2021. No missing values were imputed. Subgroup analysis looked at survival outcomes stratified by molecular characteristics, albeit with very limited samples sizes.

### Blood Sampling and Pharmacokinetics

Concentrations of etoposide were quantified in blood samples collected from a central line pretreatment, mid-infusion, at the end of drug infusion, and 1, 3, and 5 hours after the end of infusion. Additional samples were taken immediately following the end of etoposide infusion on days 2 and 3 of treatment. All drug infusion and sampling times were accurately recorded, and all samples were collected from a different lumen to that used for etoposide administration. Whole blood samples were collected in heparinized tubes and plasma was immediately obtained by centrifugation at 1200*g* for 10 minutes at 4°C. These samples were stored at −20°C prior to shipment to the Newcastle Cancer Centre Pharmacology Group (NCCPG) for analysis.

Etoposide concentrations were quantified using an API 2000 LC/MS/MS with analyst software (Applied Biosystems, CA, USA) following extraction from plasma samples with ethyl acetate as previously described.^31^ Concentrations of etoposide were quantified in patient samples utilizing a standard curve of 0.20-10.0 µg/mL, over which range calibration plots were linear, and the assay exhibited intra- and inter-assay coefficients of variation of <15%. Etoposide plasma PK parameters on day 1 of treatment were determined by non-compartmental analysis using Phoenix WinNonlin Version 8.

## Results

**Figure 1.**
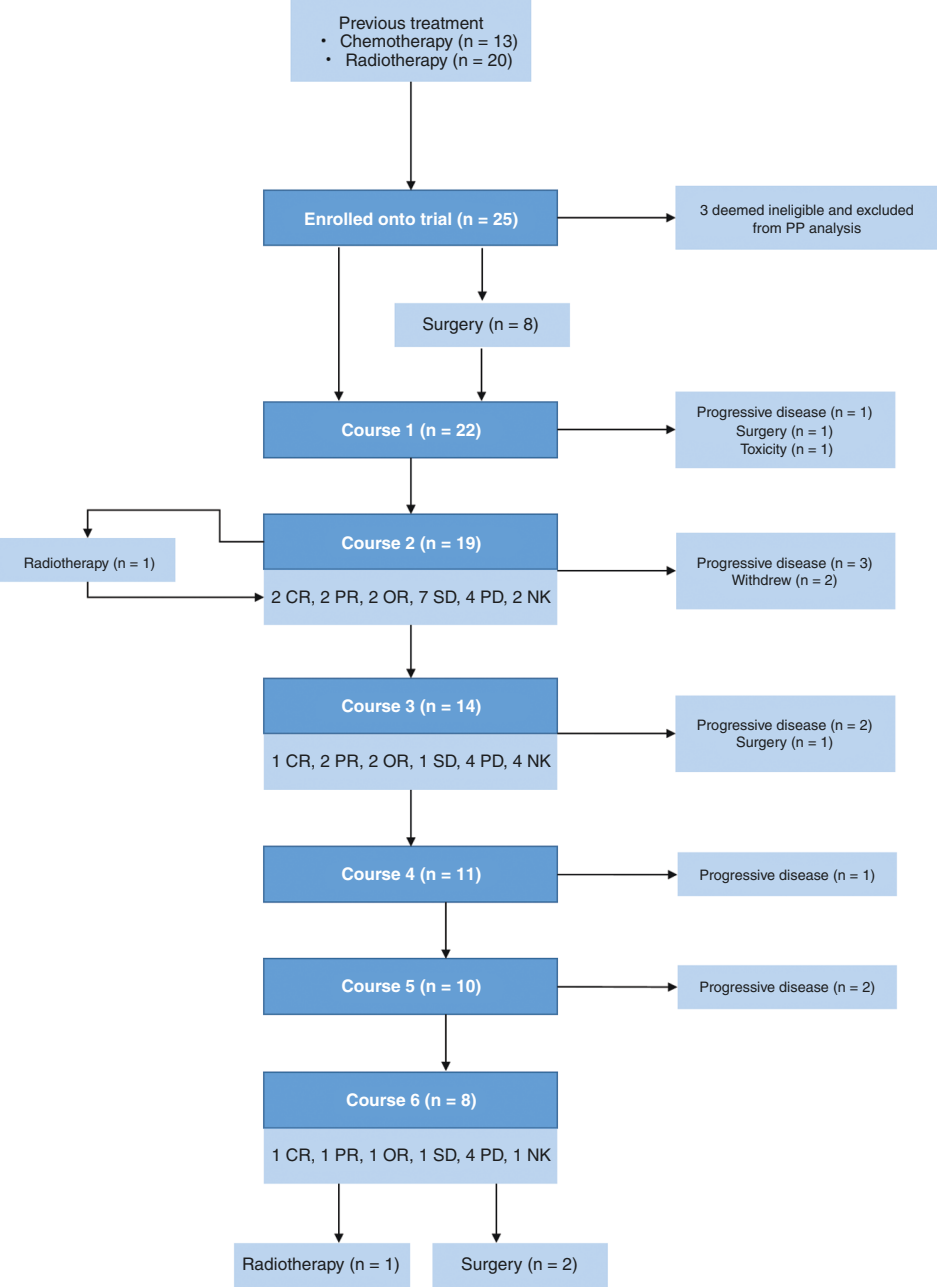
Consort diagram outlining patient eligibility and treatments received. Abbreviations: CR, complete response; PR, partial response; OR, objective response; SD, stable disease; PD, progressive disease; NK, not known.

Twenty-five patients were registered for the trial over an 8-year period (2002-2010) from 12 hospitals across the United Kingdom and Ireland. All started at least one dose of etoposide chemotherapy (Figure 1). Baseline characteristics are presented in Table 1. The primary site of 12 were infra-tentorial, 8 supra-tentorial, 1 spinal (4 metastatic, primary site unknown). Thirteen patients had previously received chemotherapy (4 as per SIOP Ependymoma 1999 trial [including etoposide], 8 as per CNS 9204 protocol [did not include etoposide], and 1 as per a pineoblastoma protocol). Twenty patients had received previous radiotherapy. Three patients were subsequently found to be ineligible for the following reasons: the first patient had a primary spinal myxopapillary ependymoma, for a second patient the diagnosis was later revised to high-grade glioma, and a third patient where the intention was to use the trials treatment as “rescue” therapy for primary progression, rather than relapse as per-protocol (PP). This patient was subsequently classified as a choroid plexus carcinoma on methylation array, in concordance with a further histopathological review. Therefore, while all 25 patients are included in the intention-to-treat (ITT) population, 22 eligible patients are included in the PP population (Figure 1). Plasma samples for the PK study were obtained from 10 patients.

**Table 1.** Baseline Patient Characteristics

Characteristic		All Patients (ITT) (N = 25)	Per-Protocol Patients (N = 22)
Age (years)	Median (min, max)	7 (2, 17)	7 (2, 17)
Sex	Female	12 (48)	11 (50)
	Male	13 (52)	11 (50)
Diagnosis	Anaplastic Ependymoma (grade III)	14 (56)	14 (64)
	Classic ependymoma (grade II)	2 (8)	2 (9)
	Ependymoma (not otherwise specified)	6 (24)	5 (23)
	Ependymoma grade I	1 (4)	1 (5)
	Myxopapillary ependymoma	1(4)	0 (0)
	High-grade glioma	1 (4)	0 (0)
Primary tumor site at registration^a^	Supra-tentorial	8 (32)	6 (27)
	Infra-tentorial	12 (48)	12 (55)
	Spine	1 (4)	0 (0)
	Unknown	4 (16)	4 (18)
Relapse site	Primary/local	16 (64)	15 (68)
	Metastatic	8 (32)	7 (32)
	Unknown	1 (4)	0 (0)
Previous tumor treatment	Chemotherapy	13 (52)	13 (59)
	Radiotherapy^b^	20 (80)	18 (82)

Data are N (%) unless otherwise specified.

^a^Four patients, all of which were metastatic, primary site was not known.

^b^Five patients who did not receive initial radiotherapy were all 5 years old or younger.

Of the 12 infra-tentorial cases, DNA methylation array data were available in 6 cases, all of which were classified as posterior fossa A subtype (PFA). Of the 6 supra-tentorial cases within the PP analysis, 2 classified as a ZFTA-fusion (previously referred to as REL-A) subtype, and no methylation data were available on the other 4 cases. Where the primary site was unknown, 2 cases were classified as PFA and no methylation data were available on the other cases.

Eight patients underwent surgery within 4 weeks of registering for the trial, after which all patients still had at least 1 area of evaluable disease.

### Treatment Received

**Figure 2.**
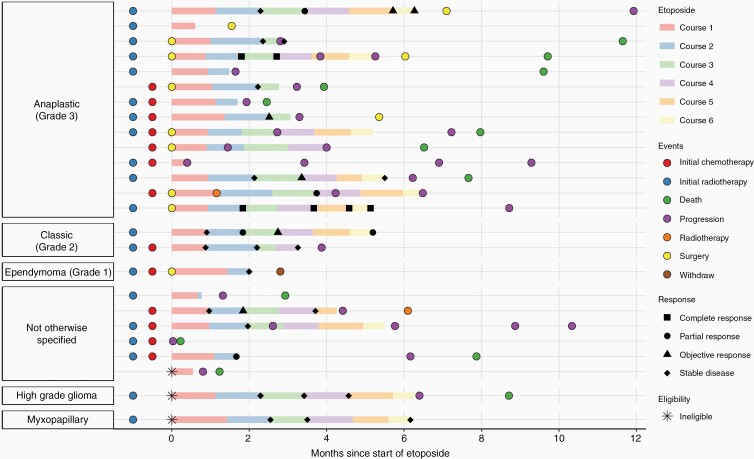
Swimmer plot showing treatment course, response, and significant events for each patient in the 12 months following the start of etoposide.

In total, 97 courses were initiated. Figure 1 and Supplementary Table 1 show the number of patients starting each course of chemotherapy, and Figure 2 shows the treatment received, response, and outcome for each patient in the 12 months following initiation of etoposide. One patient received only a single dose of etoposide as they were transferred to intensive care due to aspiration and died 1 week later. This patient is included in the ITT and PP populations.

Four patients underwent surgery following chemotherapy, in none of these patients, was it possible to achieve complete resection. One patient received 1 cycle, 1 patient received 3 cycles, and 1 patient received 6 cycles before each undergoing a partial resection. One patient received all 6 courses of etoposide before, in turn undergoing surgery, however, no tissue was removed due to the “extent of local invasion” and “multiple site disease.” Two patients received radiotherapy: 1 patient, who had not previously had radiotherapy, had radiotherapy during their second course of etoposide, while the other after discontinuing etoposide due to progression. All patients undergoing both surgery and radiotherapy are included in both ITT and PP populations.

### Response

The patient who received only one dose of etoposide was incompletely imaged according to protocol, and thus response evaluation was not possible. Central radiological review was available in 72% of post-chemotherapy scans. Each patient’s responses are presented in Figure 2 and response after each course in Supplementary Table 2.

In the ITT population, after 3 courses of etoposide, 3 patients (12%, 95% CI: 4%-30%) had achieved CR or PR, while 5 patients (20%, 95% CI: 9%-39%) achieved CR, PR, or OR (Supplementary Table 2).

In the PP population, after 3 courses of etoposide, 3 patients (14%, 95% CI: 5%-33%) achieved CR or PR, while 5 patients (23%, 95% CI: 10%-43%) achieved CR, PR, or OR (Supplementary Table 2).

In the ITT population, 6 patients (24%, 95% CI: 11%-43%) had a best overall response of CR or PR, while 9 patients (36%, 95% CI: 20%-55%) had a best overall response of CR, PR, or OR (Supplementary Table 3).

In the PP population, 6 patients (27%, 95% CI: 13%-48%) achieved a best overall response of CR or PR, while 9 patients (41%, 95% CI: 23%-61%) had a best overall response of CR, PR, or OR (Supplementary Table 3).

Of the 4 patients to previously receive etoposide, the best overall response was PR in 1 patient and OR in another, with the remaining two having PD.

### Overall Survival and Progression-Free Survival

In the ITT population, 22 patients have progressed and subsequently died, of the remaining 3 patients, 1 was lost to follow-up 4 years after trial entry, and 2 were still alive at last follow-up, 11 and 12 years after trial entry, respectively.

At 1 year, PFS was 24% (95% CI: 12%-48%) and at 3 years was 16% (95% CI: 7%-39%). Median PFS was reached at 0.5 years (95% CI: 0.39-0.83).

At 1 year, OS was 56% (95% CI: 40%-79%) and at 5 years was 20% (95% CI: 9%-44%). Median OS was 1.8 years (95% CI: 0.8-3.2).

In the PP population, 20 patients have progressed and subsequently died, the remaining 2 patients were still alive at last follow-up 11 and 12 years after trial entry. Both had previously been treated with radiotherapy, one had chemotherapy previously as per a pineoblastoma protocol and withdrew from this trial after 2 courses to have further treatment abroad (no details available). No further treatment details are available for the other case.

At 1 year, PFS was 23% (95% CI: 11%-49%) and at 3 years was 14% (95% CI: 5%-39%). Median PFS was 0.5 years (95% CI: 0.39-0.83).

At 1 year, OS was 59% (95% CI: 42%-84%) and at 5 years was 18% (95% CI: 7%-44%). Median OS was 1.9 years (95% CI: 0.9-3.2).

**Figure 3.**
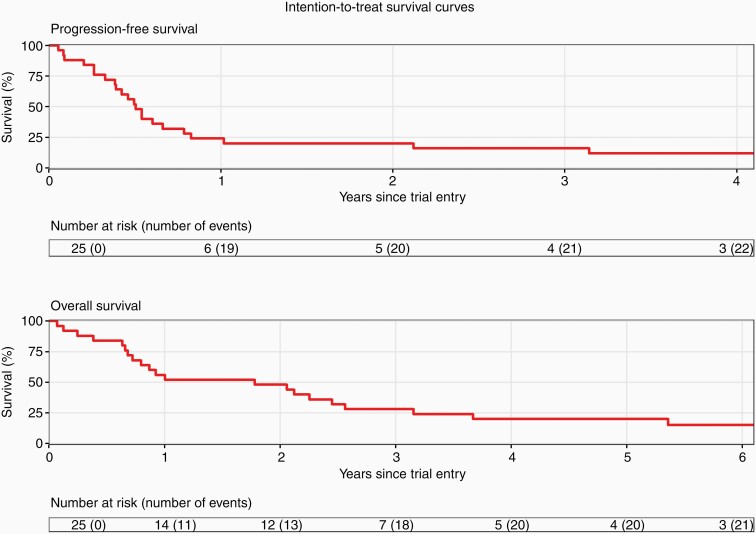
Overall and progression-free survival curves.

One patient died from aspiration during cycle 1 after administration of only one dose of etoposide but is included within this analysis. All other patients died from disease progression. Kaplan-Meier plots for OS and PFS for ITT and PP populations are presented in Figure 3 and Supplementary Figure 1, respectively.

For the 8 cases classified as PFA (all PP), median PFS was 0.4 years (95% CI: 0.3-NA) and OS was 0.9 years (95% CI: 0.6-NA). 1q gain was present in 2 of these cases, both of whose disease rapidly progressed with a PFS of only 3 months and OS of 11 and 12 months.

### Toxicity

**Figure 4.**
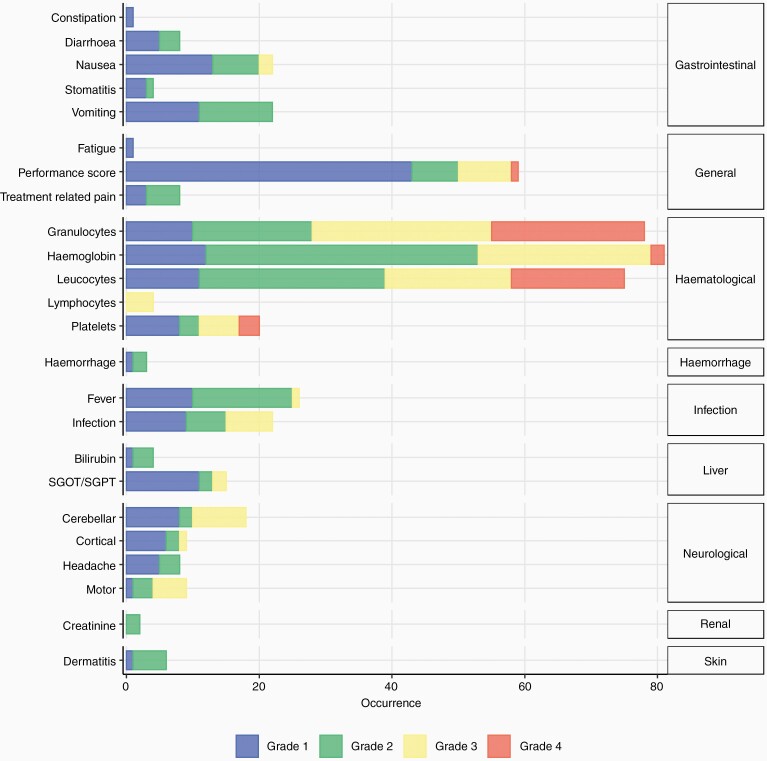
Number of adverse events recorded during chemotherapy, grouped by organ system, and colored by CTC grade.

In total, 505 adverse events (AEs) were experienced by 23 of the 25 patients. 21 (84%) patients experienced at least one grade 3 or higher AE, of which there were 162. These were predominantly hematological (51%) with 20 patients experiencing a grade 3 or higher hematological AE. AEs are detailed in Figure 4 and Supplementary Table 4, respectively.

### Pharmacokinetics

Plasma samples were obtained from a total of 10 patients at multiple time points over a 6-hour period on day 1 of treatment. Additional samples were collected immediately following the end of etoposide infusion on days 2 and 3 in 8 of these patients. All etoposide plasma concentrations measured in samples obtained following the start of etoposide infusion were above the limit of quantification for the assay.

Estimates of non-compartmental PK parameters determined on day 1 of etoposide treatment for these 11 patients are shown in Table 2. The terminal phase half-life (*T*_1/2_) of etoposide on day 1 had a median value of 2.25 hours, and a range of 1.3-5.0 hours. The median AUC_0-inf_ was 87.9 µg/mL hr (range: 70.3-143.9 µg/mL hr), *C*_max_ varied between 16.9 and 33.1 µg/mL (median: 25.35 µg/mL), and estimates of clearance ranged from 0.65 to 3.0 L/hr (median: 1.35 L/hr). Etoposide plasma concentrations measured immediately after the end of drug infusion on days 2 and 3 of treatment were comparable to those observed on day 1 in all patients where these additional samples were available for analysis. There were no clear relationships between PK parameters and disease response, though the relative low variability in PK parameters and patient numbers limited power to detect associations.

**Table 2.** Summary of Non-Compartmental Analysis of Etoposide Pharmacokinetic Data Obtained on Day 1 of Treatment

Patient	BW	BSA	*C*_max_	*T*_max_	*T*_1/2_	AUC_0-inf_	Vz	Cl
	(kg)	(m^2^)	(µg/mL)	(hr)	(hr)	(µg/mL.hr)	(L)	(L/hr)
1	15.1	0.7	30.7	1.1	2.0	101.6	1.9	0.7
2	65.0	1.8	33.1	1.0	2.0	97.2	5.2	1.8
3	53.7	1.6	26.3	1.1	2.0	82.1	5.7	1.9
4	45.5	1.4	16.9	1.2	2.8	70.3	6.1	1.5
5	48.8	1.5	24.4	1.1	5.0	143.9	7.6	1.0
6	35.7	1.2	28.5	1.0	2.5	93.6	4.6	1.3
7	103.4	2.2	22.6	1.1	3.1	72.3	13.6	3.0
8	25.6	1.0	18.6	2.4	1.7	70.4	3.4	1.4
9	14.8	0.7	31.8	1.1	1.3	85.1	1.5	0.8
10	26.9	1.0	17.2	1.3	3.2	90.7	4.9	1.1
Mean	43.5	1.3	25.0	1.2	2.6	90.7	5.5	1.4
SD	26.8	0.5	6.1	0.4	1.0	21.8	3.4	0.7
Median	40.6	1.3	25.4	1.1	2.3	87.9	5.1	1.4
Range	14.8-103.4	0.7-2.2	16.9-33.1	1.0-2.4	1.3-5.0	70.3-143.9	1.5-13.6	0.7-3.0

Abbreviations: AUC_0-inf_, area under the plasma concentration-time curve; BW, body weight; BSA, body surface area; Cl, clearance; *C*_max_, peak plasma concentration; SD, standard deviation; *T*_max_, time of peak plasma concentration; *T*_1/2_, terminal half-life; Vz, apparent volume of distribution.

## Discussion

The results of this study show evidence of a response to IV etoposide in up to 41% of patients with relapsed ependymoma, confirming its activity in this patient group.

The response rate of 41% is similar to that seen in previous observational studies using oral etoposide, and a randomized trial comparing the use of oral etoposide with erlotinib in patients with relapsed ependymoma.^18–21^ While response rates and PFS were similar, OS was longer in this trial than in previously reported studies. In this study, median PFS was 6 months and OS of 1.9 years. In Chamberlain’s study of 12 patients, all of whom had previously received chemotherapy and radiotherapy, 2 patients experienced PR to oral etoposide and 4 had stabilizing of disease.^18^ In these patients, median PFS was 7 months and the median OS was 10 months (range 6-16 months), whereas in those not responding median survival was 3 months (range 2-4 months).^18^ Sandri et al, in which half the patients were at second-fourth relapse, and all of whom had previously received chemotherapy and 10 of 12 radiotherapy, reported a response rate of 41% to oral etoposide, with median PFS of 6 months, 2-year PFS of 16.7% and median OS of 7 months.^19^ Jakacki et al reported a response (PR or minor response) in 3 of 12 patients to oral etoposide with median PFS of only 65 days in 12 patients, all of whom had previously received radiotherapy and had a median of 2 relapses.^21^ As these are historical comparisons, it is not possible to draw any definitive conclusion on a true difference in activity of IV vs oral administration of etoposide, or how much any differences in OS are influenced by differences in patient populations. A recent retrospective analysis of 186 cases of relapsed ependymoma highlighted, how at each subsequent relapse, the prognosis of relapsed ependymoma decreases, related, in part, to the prior therapy.^5^ The impact of previous etoposide exposure in 4 of the patients in this cohort is not possible to fully assess.

Similarly, the outcome of patients with ependymoma is increasingly recognized as related to the biological characteristics of their underlying tumors, including, broad biological subgrouping and specific biomarkers such as 1q gain.^32–34^ In this study, limited biological data were available for a subset of patients, and while the low numbers precluded investigation of association with radiological response or patient outcome, it is notable that patients with both a PFA subtype and 1q gain progressed and died rapidly. Additionally, and perhaps unsurprisingly, the only DNA methylation groups represented in this cohort were those known to be associated with poor prognosis.^32^

Currently, this, often poorly characterized, clinical and biological heterogeneity makes it difficult to assess the relative merits of different treatment strategies. Accounting for these patients, previous treatment and biological factors will be critical for the next generation of trials in relapsed ependymoma, particularly as rationally targeted biological agents are developed, which may be of relevance to particular patient populations. This will require international studies and collaboration to achieve adequate statistical power. Thorough data collection of patients across their disease journeys, through sequential relapses and response to serial therapies, will also ensure that we can learn as much as possible from each and every patient. Similarly, the involvement of patients and parents in designing studies and the collection of patient outcome measures will ensure that futures studies adequately address the needs of patients with this challenging tumor.

The results of this study show that the treatment with IV etoposide was generally well tolerated, with hematological toxicity the main AEs. These were more common, though of similar type, to those seen in the studies of oral etoposide.^18–21^

The PK of etoposide were investigated in 10 study patients, showing comparable PK properties to previously published studies.^31,35,36^ A linear relationship was observed between body weight and etoposide clearance as would be anticipated in a pediatric study. Interestingly, a large volume of distribution was observed for patient 7, who had a BW >100 kg and BSA >2 m^2^, as would be predicted for a highly lipophilic drug. Compared to oral etoposide, the IV dosing achieved an overall lower total AUC per 28-day cycle.^37,38^ However, this may be offset by a higher *C*_max_ and differences in blood-brain/tumor barrier penetration, as some studies have suggested higher concentrations in tumors following IV compared to oral administration.^37–39^

Over time the role of surgery at relapse has become clearer. When feasible and likely to achieve a complete or near-total resection with acceptable risks of neurosurgical morbidity, then there is a clear but difficult to quantify survival benefit.^5^ In the modern era, multidisciplinary team discussion within units and even at a national level has tended to encourage a higher rate of reoperation in ependymoma.^40,41^ At relapse, however, surgeons may be more willing to consider further surgery if an additional systemic treatment is also proposed to consolidate its effect.^42^ It is worth noting that in this trial, patients were only eligible if their tumors were not suitable for complete resection, and that chemotherapy did not facilitate complete resection in any of the patients enrolled.

In summary, this study confirms some activity of IV etoposide against relapsed ependymoma, however, this is modest and was not sustained, similar to that observed with oral etoposide, but with increased toxicity. The results here confirm the dismal prognosis in this disease but add to the rationale to include etoposide within drug combinations developed for ependymoma. Subsequent to this study being undertaken, there has been an increased use of re-irradiation for relapsed ependymoma, which while for many slowing disease course, does not for most patients result in long-term survival.^5,6^ It is likely that to transform the outcome of patients with relapsed ependymoma, novel treatment strategies and combinations will be required and to be developed, and tested within international trials. Despite the poor prognosis, it is notable that there are currently no ongoing trials specifically for this patient population.

## Supplementary Material

vdac053_suppl_Supplementary_Material
