# Supplementary material for: Phase II study of intravenous etoposide in patients with relapsed ependymoma (CNS 2001 04)
Source: Neurooncol Adv. 2022 Apr 13;4(1):vdac053. doi: 10.1093/noajnl/vdac053 (PMC9113139; doi:10.1093/noajnl/vdac053)
Supplement: vdac053_suppl_Supplementary_Material [file vdac053_suppl_supplementary_material.docx]

**Supplementary Material**

*Supplementary Figure 1: Overall and Progression-Free Survival Curves for Per Population Analysis*

**
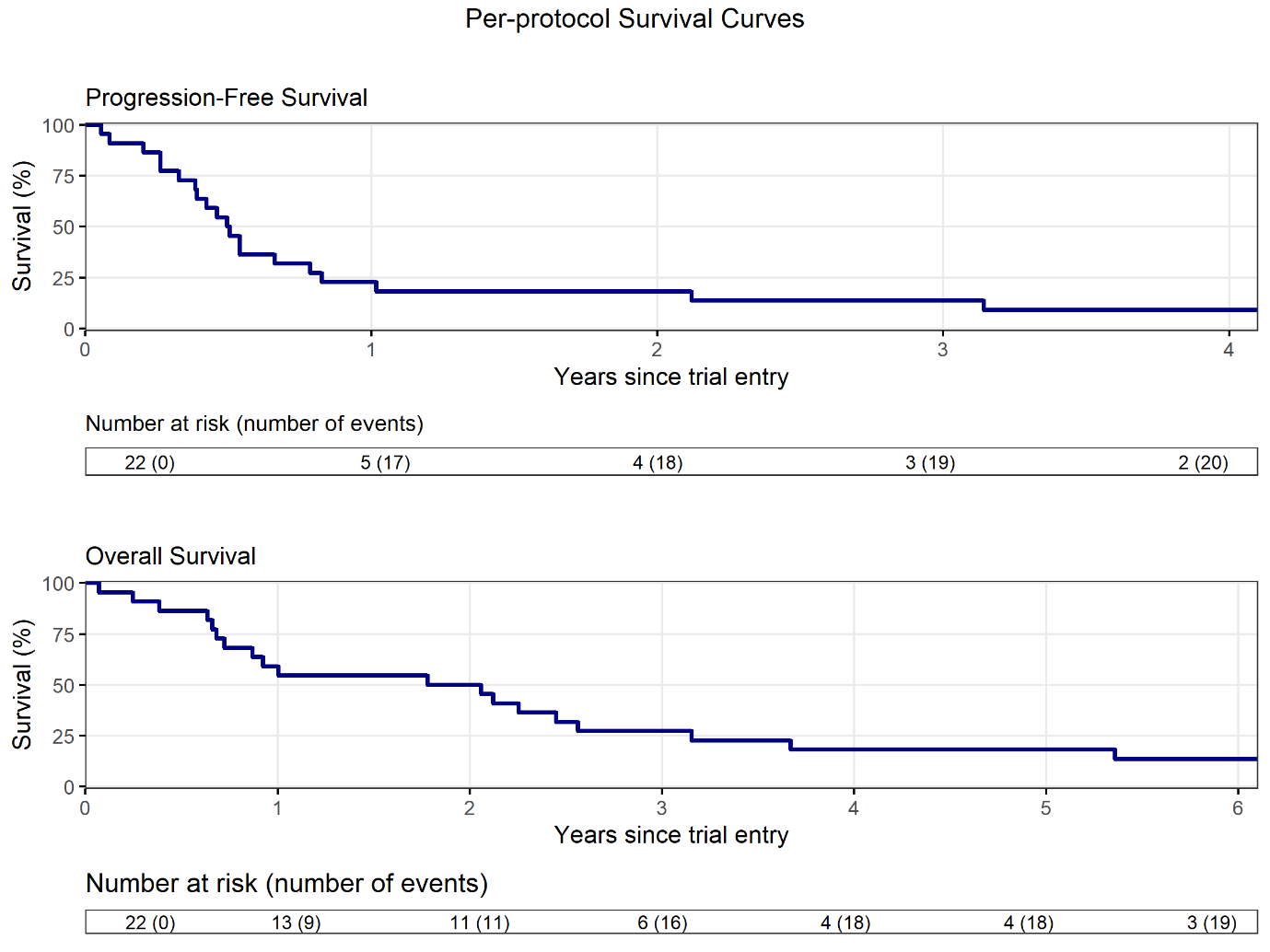
**

***Supplementary Table 1: Patients Starting Each Chemotherapy Course***

| Etoposide Chemotherapy Course | Patients Starting Course | |
| --- | --- | --- |
|  | **ITT**  **(*N* = 25)** | **PP**  **(*N* = 22)** |
| 1 | 25 (100) | 22 (100) |
| 2 | 21 (84) | 19 (86) |
| 3 | 16 (64) | 14 (64) |
| 4 | 13 (52) | 11 (50) |
| 5 | 12 (48) | 10 (45) |
| 6 | 10 (40) | 8 (36) |

*Data are N (%). ITT, Intention to Treat; PP, Per Protocol.*

***Supplementary Table 2: Response by Course***

| **Response** | **Intention to treat**  **(N = 25)** | | | **Per protocol**  **(N = 22)** | | |
| --- | --- | --- | --- | --- | --- | --- |
|  | **Course 2** | **Course 3** | **Course 6** | **Course 2** | **Course 3** | **Course 6** |
| Complete Response | 2 (8) | 1 (4) | 1 (4) | 2 (9) | 1 (5) | 1 (5) |
| Partial Response | 2 (8) | 2 (8) | 1 (4) | 2 (9) | 2 (9) | 1 (5) |
| Objective Response | 2 (8) | 2 (8) | 1 (4) | 2 (9) | 2 (9) | 1 (5) |
| Stable Disease | 9 (36) | 3 (12) | 2 (8) | 7 (32) | 1 (5) | 1 (5) |
| Progressive Disease | 4 (16) | 4 (16) | 5 (20) | 4 (18) | 4 (18) | 4 (18) |
| Discontinued | 4 (16) | 9 (36) | 14 (56) | 3 (14) | 8 (36) | 13 (59) |
| Response not available | 2 (8) | 4 (16) | 1 (4) | 2 (9) | 4 (18) | 1 (5) |

***Supplementary Table 3: Best Overall Response to Chemotherapy***

| Best Overall Response | ITT | PP |
| --- | --- | --- |
| Complete Response | 2 (8) | 2 (9) |
| Partial Response | 4 (16) | 4 (18) |
| Objective Response | 3 (12) | 3 (14) |
| Stable Disease | 7 (28) | 5 (23) |
| Progressive Disease | 8 (32) | 7 (32) |
| Did not reach first scan | 1 (4) | 1 (5) |
| Total | 25 (100) | 22 (100) |

*Data are N (%). ITT, Intention to Treat; PP, Per Protocol.*

***Supplementary Table 4: Number of Adverse Event Occurrences by Grade.***

| Adverse Event | Grade 1 | Grade 2 | Grade 3 | Grade 4 | Total |
| --- | --- | --- | --- | --- | --- |
| Bilirubin | 1 | 3 | 0 | 0 | 4 |
| Cerebellar | 8 | 2 | 8 | 0 | 18 |
| Constipation | 1 | 0 | 0 | 0 | 1 |
| Cortical | 6 | 2 | 1 | 0 | 9 |
| Creatinine | 0 | 2 | 0 | 0 | 2 |
| Dermatitis | 1 | 5 | 0 | 0 | 6 |
| Diarrhoea | 5 | 3 | 0 | 0 | 8 |
| Fatigue | 1 | 0 | 0 | 0 | 1 |
| Fever | 10 | 15 | 1 | 0 | 26 |
| Granulocytes | 10 | 18 | 27 | 23 | 78 |
| Haemoglobin | 12 | 41 | 26 | 2 | 81 |
| Haemorrhage | 1 | 2 | 0 | 0 | 3 |
| Headache | 5 | 3 | 0 | 0 | 8 |
| Infection | 9 | 6 | 7 | 0 | 22 |
| Leucocytes | 11 | 28 | 19 | 17 | 75 |
| Lymphocytes | 0 | 0 | 4 | 0 | 4 |
| Motor | 1 | 3 | 5 | 0 | 9 |
| Nausea | 13 | 7 | 2 | 0 | 22 |
| Performance Score (Lanksy/Karnofsky) | 43 | 7 | 8 | 1 | 59 |
| Platelets | 8 | 3 | 6 | 3 | 20 |
| AST/ALT* | 11 | 2 | 2 | 0 | 15 |
| Stomatitis | 3 | 1 | 0 | 0 | 4 |
| Treatment Related Pain | 3 | 5 | 0 | 0 | 8 |
| Vomiting | 11 | 11 | 0 | 0 | 22 |
| Total | 174 | 169 | 116 | 46 | 505 |

* aspartate transaminase/alanine transaminase
